# Computed Tomography Radiomics-Based Prediction of Microvascular Invasion in Hepatocellular Carcinoma

**DOI:** 10.3389/fmed.2022.819670

**Published:** 2022-03-24

**Authors:** Wenjun Yao, Shuo Yang, Yaqiong Ge, Wenlong Fan, Li Xiang, Yang Wan, Kangchen Gu, Yan Zhao, Rujing Zha, Junjie Bu

**Affiliations:** ^1^Department of Radiology, The Second Affiliated Hospital of Anhui Medical University, Hefei, China; ^2^Department of Radiology, Anhui Mental Health Center, Hefei, China; ^3^GE Healthcare China, Shanghai, China; ^4^Department of Hematological Lab, The Second Affiliated Hospital of Anhui Medical University, Hefei, China; ^5^School of Biomedical Engineering, Anhui Medical University, Hefei, China; ^6^Department of Radiology, Division of Life Science and Medicine, The First Affiliated Hospital of USTC, School of Life Science, University of Science and Technology of China, Hefei, China; ^7^The Fourth Affiliated Hospital of Anhui Medical University, Hefei, China

**Keywords:** hepatocellular carcinoma, microvascular invasion, radiomics, nomogram, triphasic computed tomography

## Abstract

**Background:**

Due to the high recurrence rate in hepatocellular carcinoma (HCC) after resection, preoperative prognostic prediction of HCC is important for appropriate patient management. Exploring and developing preoperative diagnostic methods has great clinical value in treating patients with HCC. This study sought to develop and evaluate a novel combined clinical predictive model based on standard triphasic computed tomography (CT) to discriminate microvascular invasion (MVI) in hepatocellular carcinoma (HCC).

**Methods:**

The preoperative findings of 82 patients with HCC, including conventional clinical factors, CT imaging findings, and CT texture analysis (TA), were analyzed retrospectively. All included cases were divided into MVI-negative (*n* = 33; no MVI) and MVI-positive (*n* = 49; low or high risk of MVI) groups. TA parameters were extracted from non-enhanced, arterial, portal venous, and equilibrium phase images and subsequently calculated using the Artificial Intelligence Kit. After statistical analyses, a clinical model comprising conventional clinical and CT image risk factors, radiomics signature models, and a novel combined model (fused radiomic signature) was constructed. The area under the curve (AUC) of the receiver operating characteristics (ROC) curve was used to assess the performance of the various models in discriminating MVI.

**Results:**

We found that tumor diameter and pathological grade were effective clinical predictors in clinical model and 12 radiomics features were effective for MVI prediction of each CT phase. The AUCs of the clinical, plain, artery, venous, and delay models were 0.77 (95% CI: 0.67–0.88), 0.75 (95% CI: 0.64–0.87), 0.79 (95% CI: 0.69–0.89), 0.73 (95% CI: 0.61–0.85), and 0.80 (95% CI: 0.70–0.91), respectively. The novel combined model exhibited the best performance, with an AUC of 0.83 (95% CI: 0.74–0.93).

**Conclusions:**

Models derived from triphasic CT can preoperatively predict MVI in patients with HCC. Of the models tested here, the novel combined model was most predictive and could become a useful tool to guide subsequent personalized treatment of HCC.

## Introduction

Hepatocellular carcinoma (HCC), one of the most common malignant tumors, currently ranks as the second most common cause of cancer-related deaths worldwide, with more than half of all HCC cases diagnosed in China (1). HCC's high potential for vascular invasion, metastasis, and recurrence after resection explains its poor prognosis. Previous studies have indicated that microvascular invasion (MVI) is one of the most important factors influencing HCC recurrence and the prognosis of patients with this tumor (2, 3).

Several therapies such as liver resection (LR), liver transplantation, and transcatheter arterial chemoembolisation (TACE) are conventionally used to treat HCC. LR is the most common therapeutic option, while TACE is recommended for patients without vascular invasion or extrahepatic metastasis (4).

In most cases, aspiration biopsy is the first step in determining whether the tumor is benign or malignant after clinical and imaging evaluation. Clinicians then assess clinical symptoms, specific serological indicators, and pathological results to determine treatment decisions. To date, MVI has often been overlooked in these initial steps, as an aspiration biopsy does not produce sufficient tissue samples to evaluate MVI; instead, aspiration biopsy is only capable of differentiating benign from malignant tumors and grading tumors according to the Edmondson-Steiner (E-S) scale. Thus, preoperative reliable prognostic markers for patient stratification are needed.

Interest in presurgical imaging for MVI assessment in patients with HCC has grown significantly in recent years. Conventional imaging-based assessments, including tumor density, shape, and enhancement, have not been capable of predicting MVI. However, previous studies have indicated that imaging features (5, 6) such as tumor margin and size, radiological capsule, locally convex nodules, multinodular fusion, intratumoural artery and low-density signs (also called two-trait predictor of venous invasion, or TTPVI), as well as portal vein tumor thrombus (PVTT), can be used to predict MVI. However, these parameters can be easily influenced by interobserver variability and were not considered quantitative features.

Radiomics is an emerging field that permits the extraction of high-dimensional information from medical images and is considered to reflect tissue heterogeneity (7–11). Thus, the radiomics signature, which comprises multiple texture features, has become a powerful prognostic biomarker. This signature augments available clinical data and has been a significant predictor of clinically relevant factors. According to the literature, by constructing appropriate models with radiomics features, researchers have achieved successful assessment and prediction abilities in various challenging clinical tasks (12). Studies have shown that computed tomography (CT) texture analysis can be used for MVI prediction, and that predictions derived from CT-based nomograms are more accurate than other predictive models (13, 14).

Due to HCC's high recurrence rate after resection, preoperative prognostic prediction of HCC is important for appropriate patient management. Therefore, exploring and developing preoperative diagnostic methods has the potential for great clinical value in treating patients with HCC. With this study, we thus aimed to construct MVI-specific diagnostic models by analyzing clinical and imaging features, as well as texture analysis (TA)-derived image parameters based on standard triphasic CT. We also sought to evaluate these models' diagnostic performance to explore whether models based on standard triphasic CT could preoperatively discriminate MVI in HCC.

## Methods

### Study Design and Patient Population

This was a single-center retrospective study approved by the Institutional Review Board of the Second Affiliated Hospital of Anhui Medical University [No. YX2020-101 (F1)], which waived the need for individual informed consent; this study complies with the Declaration of Helsinki (as revised in 2013).

Participants with confirmed HCC by histopathology and who underwent CT examination before surgery were included in our study between December 2018 and September 2021. Relevant demographic and clinical patient information was retrieved from the electronic medical record. The inclusion criteria were as follows: (1) complete medical information; (2) CT examination using the same equipment and scanning parameters; (3) CT examination comprising non-enhanced, arterial, portal venous, and equilibrium phase scans; and (4) confirmation of HCC by pathology with MVI evaluation. The exclusion criteria were as follow: (1) metastatic or recurrent tumor; (2) liver transplantation; (3) poor CT image quality interfering with the observation, such as breathing or motion artifacts; (4) incomplete CT images; and (5) pathological images not meeting the criteria and/or MVI not evaluated.

A total of 82 participants over 18 years old (65 men and 17 women) were included in our study. Demographic data, serum alpha-fetoprotein (AFP) levels, and history of hepatitis B infection were collected and recorded.

### Histological Analysis

Histopathologic features, including pathology results, E-S grade, MVI status, and liver cirrhosis around the tumor were evaluated by two pathologists with 5 years and 8 years of professional experience, respectively. If a difference of opinion arose, the two doctors reached a final conclusion through discussion. MVI was defined as when tumor cell nests could be observed under microscopy in the vascular space lined by endothelium. There are three additional subgrades based on the distance factor: M0, no MVI; M1 (low-risk group), ≤5 MVI in the liver tissue adjacent to the tumor ( ≤1 cm); and M2 (high-risk group), >5 MVI or MVI in liver tissue adjacent to the tumor (>1 cm). Finally, we divided patients with M0 into the MVI-negative group and patients with M1/M2 into the MVI-positive group.

### CT Scan and Image Analysis

CT examinations were performed using a Philips iCT 256-slice Brilliance CT scanner (Philips Healthcare, Cleveland OH, USA). Patients were required to fast, with no intake of solid foods or liquids for >4 h prior to scanning; they were allowed to drink 800 mL of water immediately before the examination. All participants underwent non-enhanced and standard triphasic scans. A non-ionic contrast agent at a dose of 1.5 ml/kg and a flow rate of 3.0 ml/s using a high-pressure syringe was injected into the right cubital vein after completing the non-enhanced scan. The acquisition time of standard triphasic images was 30, 60, and 120 s after contrast material injection, respectively. Scanning parameters were as follows: tube voltage, 120 kV; tube current, 230 mAs; slice thickness, 5 mm; slice interval, 0 mm; and pitch ratio, 0.6.

**Figure 1:**
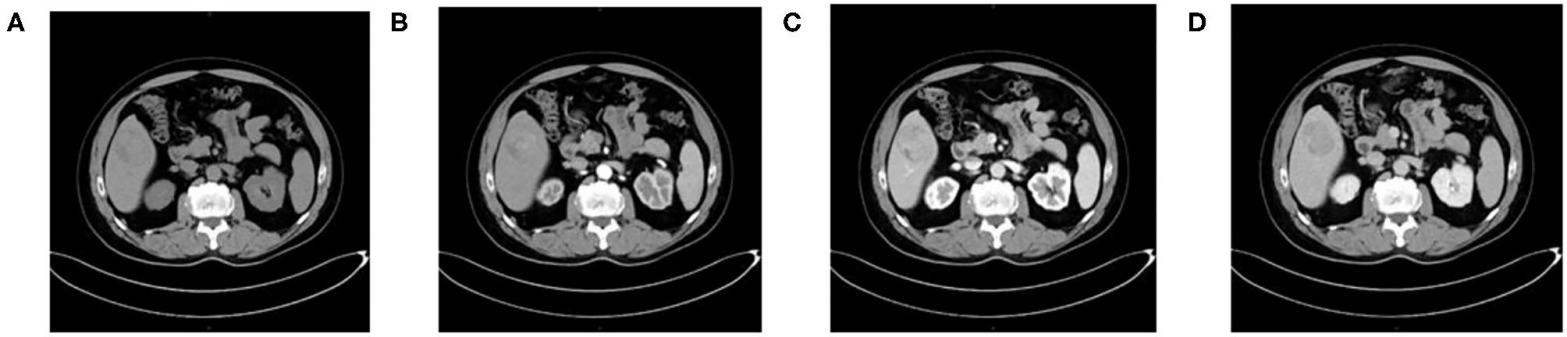
A 54-year-old man with HCC who is MVI-negative. **(A)** Non-enhanced phase showing a low-isodense tumor in the hepatic segment V, **(B)** arterial phase, **(C)** portal venous phase, and **(D)** equilibrium phase showing smooth tumor margin and TTPVI.

**Figure 2:**
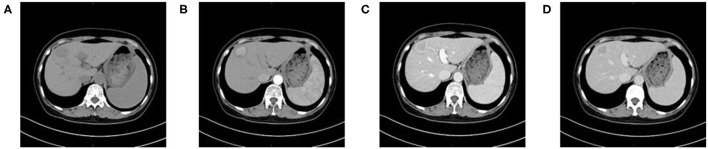
A 59-year-old woman with HCC who is MVI-positive. **(A)** Non-enhanced phase showing a low-density tumor in the hepatic segment IV, **(B)** arterial phase showing a hyper-vascular tumor with obvious enhancement, **(C)** portal venous phase, and **(D)** equilibrium phase display non-smoothed tumor margin and TTPVI.

Images collected from the participants were retrieved from the hospital's Picture Archiving and Communication System (PACS). Two radiologists with 14 and 13 years of experience in abdominal imaging reviewed these images separately and were blinded to the pathological results (Figures 1A–D, 2A–D). The more senior of the two radiologists made the final decision if a difference of opinion arose. The evaluated image features included: (1) tumor margin: when using a narrow window setting to observe, the margin was considered to be smooth if more than 90% edge of the entire tumor was “pencil-thin” sharp on arterial, portal venous, and equilibrium phase images (0, smooth; 1, non-smooth); (2) diameter: the maximal diameter of the largest cross section of the tumor; (3) capsule: a hypodense halo encircling the tumor (0, absent; 1, incomplete; 2, complete); (4) locally convex nodules (0, the nodule separated by normal liver parenchyma; 1, the nodule in direct contact with the surface capsule of liver); (5) multinodular fusion (0, absent; 1, present); (6) TTPVI: two-trait predictor of venous invasion including intratumoural artery and low-density signs (0, absent; 1, present); (7) PVTT: filling defect with enhancement in the portal or hepatic vein observed at the portal venous phase (0, absent; 1, present). For multifocal HCC, the largest focus was selected for analysis.

### Texture Features

**Figure 3:**
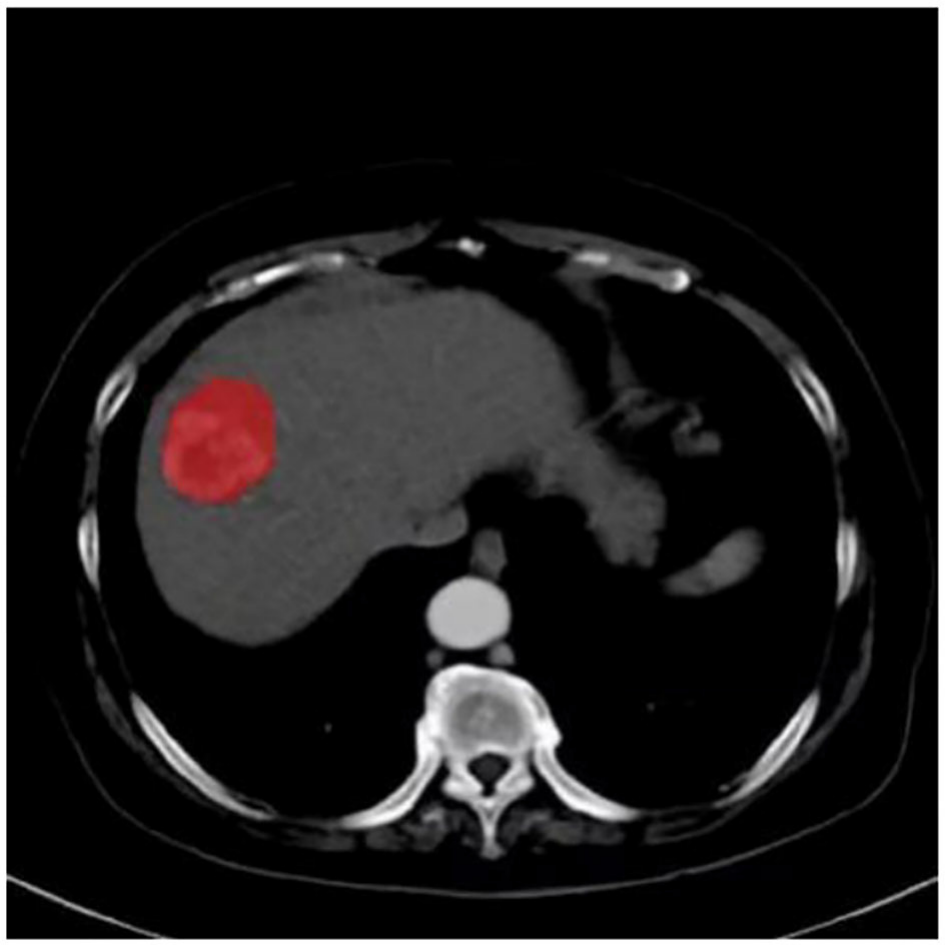
Lesion segmentation for radiomics analysis.

For all study participants, texture features were extracted from each of the four CT imaging phases noted above. Preoperative CT images of all participants were exported in Digital Imaging and Communications in Medicine (DICOM) format. Images showing lesions were then used for manual region-of-interest (ROI) delineation (Figure 3). Two radiologists with 14 and 13 years of experience in CT imaging used an open-source software program (ITK-SNAP, V3.3) (15) to delineate the largest cross-sectional HCC area on the images of the four phases. For multifocal HCC, the largest tumor was selected for analysis. To assess intra-observer precision, the data were collected twice by the same radiologist with a 2-week interval; furthermore, inter-observer precision was assessed by measurements independently performed by two radiologists. The intraclass correlation coefficient (ICC) was applied to analyze the inter-observer and intra-observer agreements of the feature extraction.

The Artificial Intelligence Kit Version (V3.3.0 GE Healthcare, Shanghai, China) which complied to IBSI (Image Biomarker Standardization Initiative) was used to extract texture features. Feature types including the first-order, shape, Gray Level Cooccurrence Matrix (GLCM), Gray Level Run Length Matrix (GLRLM), Gray Level Size Zone Matrix (GLSZM), Neighboring Gray Tone Difference Matrix (NGTDM), and Gray Level Differential Matrix (GLDM), as well as transform including wavelet and Laplacian of Gaussian (Log) were extracted based on ROIs after input of all DICOMS and ROI lesion images into the software. Overall, 1,317 texture features were extracted per ROI.

### Features Selection and Model Construction

#### Clinical Model

Clinical and imaging characteristics analysis was performed as follow steps: (1) chi-square test or Fisher's exact test was used for nominal variables while continuous variables which complied to abnormal distribution was analyzed by Mann–Whitney *U*-test to filter characters which *p* < 0.05; (2) significant features were analyzed by using univariate logistic regression and features with *p* < 0.05 were enrolled in multivariate logistic regression to construct the clinical model, meanwhile, feature with Variance Inflation Factor (VIF) > 10 was eliminate to solve the problem of multicollinearity.

#### Radiomics Signature Model

Features from each phase of non-enhanced, arterial, portal venous, and equilibrium were analyzed independently through following steps: (1) features with both inter- and intra-observer ICCs > 0.75 were retained; (2) Mann-Whitney *U*-test was used to explore whether the features were significantly different between the two groups (*p* < 0.05), then significant different features were furtherly analyzed by univariate logistic regression to explore whether the features were discriminative between the two groups (*p* < 0.05); (3) the minimum redundancy and maximum relevance (MRMR) method was used to eliminate redundancy meanwhile retain features most relevant to the MVI; (4) finally, backward stepwise multivariable logistic regression was used to select the final features and construct the regression model, features with *p* < 0.05 were independent risk predictors. Considering the small number of available datasets, there was no data grouping in this study. However, 10-fold cross validation with 10 times repetition was applied to prove that the model was feasible in distinguishing one group from another and that the result was not due to overfitting. Lastly, Delong test was used to compare the performance of the two models (*p* < 0.05).

#### Combined Model

The scores obtained from the clinical model and from the four radiomics models were incorporated into a backward stepwise multivariate logistic regression analysis. Scores from arterial and venous were finally retained and the combined model was constructed, furthermore displayed as a nomogram.

**Figure 4:**
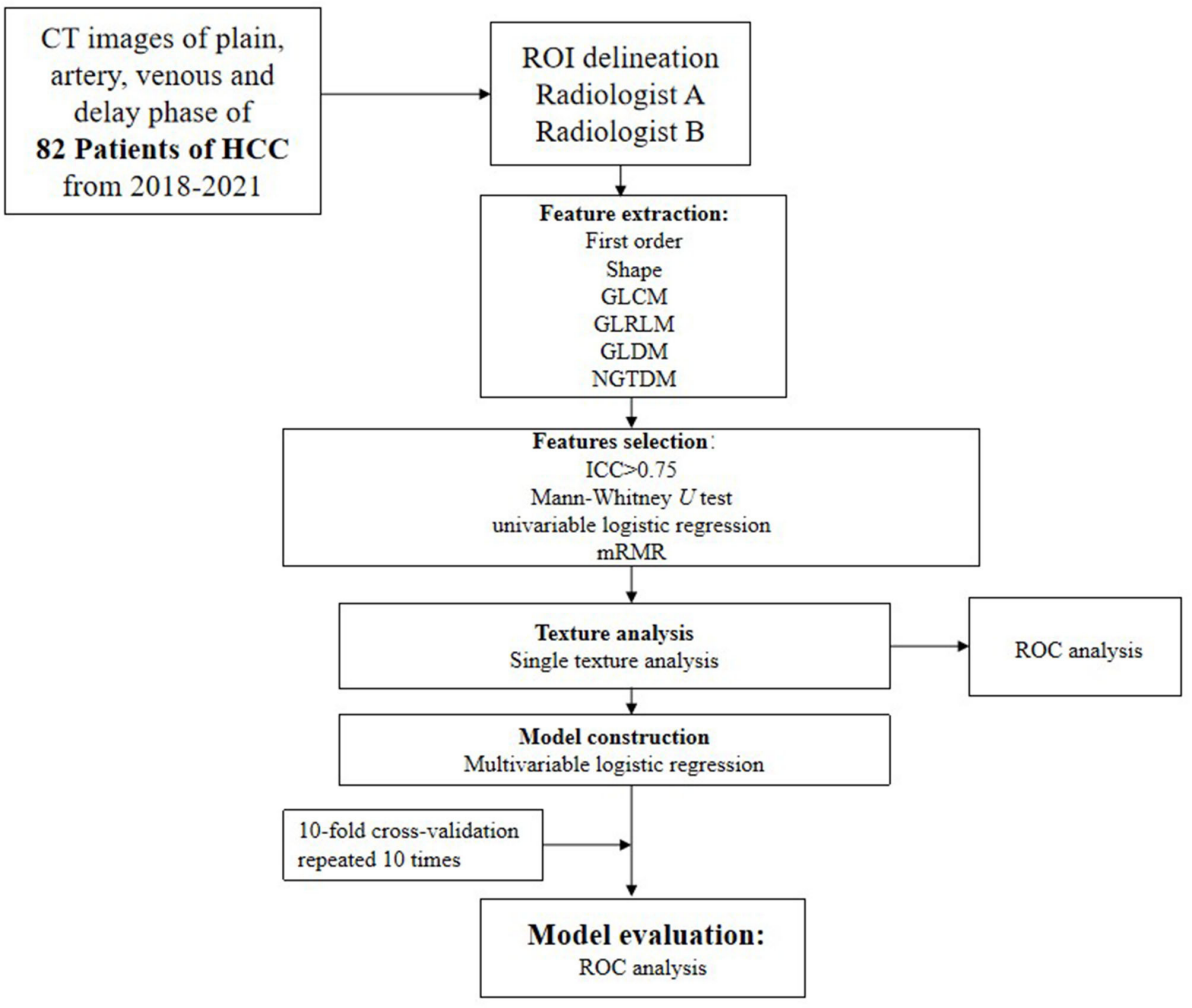
Flowchart of feature selection, texture analysis, and model construction.

Flowchart of our study is shown in Figure 4. All statistical analyses were performed using R (version 3.5.1) and Python (version 3.5.6). Statistical significance was defined as a two-tailed *p* < 0.05. Areas under the receiver operating characteristics (ROC) curves and areas under the curves (AUCs) were used to evaluate the diagnostic value of the final models.

## Results

Over all, 82 participants (65 men and 17 women) were included in our retrospective study. Of these participants, 33 were classified as MVI-negative and 49 as MVI-positive.

### Analysis of Clinical and Imaging Characteristics

The baseline clinical characteristics and CT imaging features of the MVI-negative and MVI-positive groups are summarized in Supplementary Table 1. E-S grade, serum AFP levels, tumor diameter, and multinodular fusion were significantly different between groups (*p* < 0.05).

### Texture Analysis

For each phase of CT image, features number with inter- and intra- ICCs bigger than 0.75, features number with *p* < 0.05 in Mann-Whitney *U*-test and univariate logistic are shown in Supplementary Table 2. Then MRMR was applied to eliminate feature redundancy while retaining the most predictive features. For each phase 12 features are retained. Supplementary Table 3 shows the predictive performance of each feature, and the performance of the optimal textural feature in each phase were shown in Supplementary Table 4.

### Model Construction and Evaluation

#### Clinical Model

**Figure 5:**
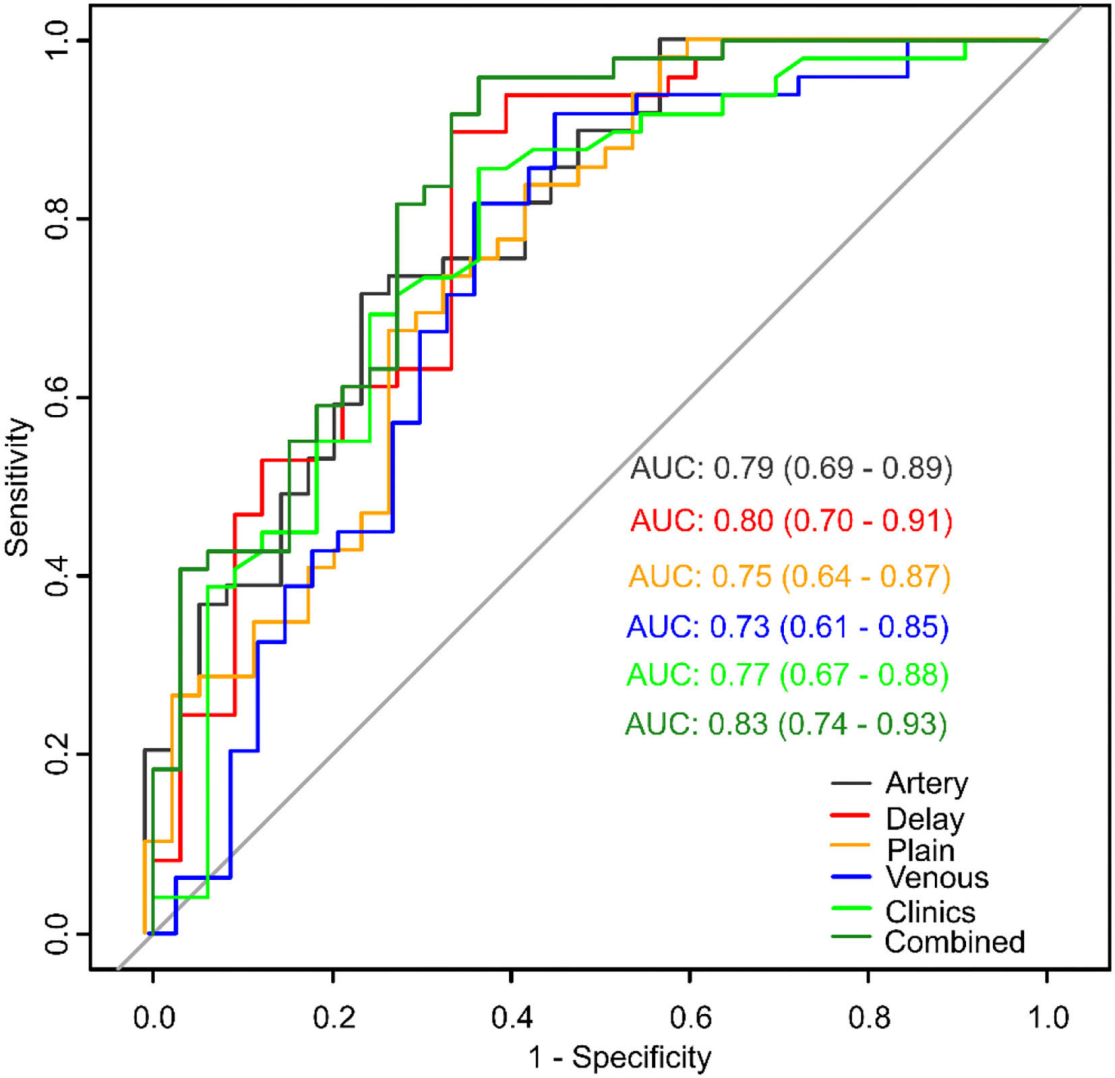
Model comparisons of receiver operating characteristics (ROC) curves for prediction of microvascular invasion.

Univariate analysis showed that diameter, multinodular fusion, E-S grade, and serum AFP level were significantly associated with MVI (*p* < 0.05). Multivariable analysis identified that diameter [odds ratio (OR) 1.21; 95% confidence interval (CI) 1.06–1.42; *p* = 0.008] and E-S grade (OR 2.21; 95% CI 1.12–4.73; *p* = 0.03) were independent predictors of MIV, and that these were also effective factors for clinical model construction. The AUC of the clinical model (Figure 5) combining the two factors was 0.77 (95% CI: 0.67–0.88).

#### Radiomics Signature Model Using Single CT Phases

Model of each CT phase was constructed by multivariate logistic regression. The four radiomics signature models were named as plain, artery, venous and delay model, which based on non-enhanced, arterial, portal venous, and equilibrium phase, respectively. Figure 5 shows the performance of each model. The AUCs of the plain, artery, venous, and delay models were 0.75 (95% CI: 0.64–0.87), 0.79 (95% CI: 0.69–0.89), 0.73 (95% CI: 0.61–0.85), and 0.80 (95% CI: 0.70–0.91), respectively. The delay model exhibited better predictive performance than did the other radiomic signature models.

#### Combined Model

**Figure 6:**
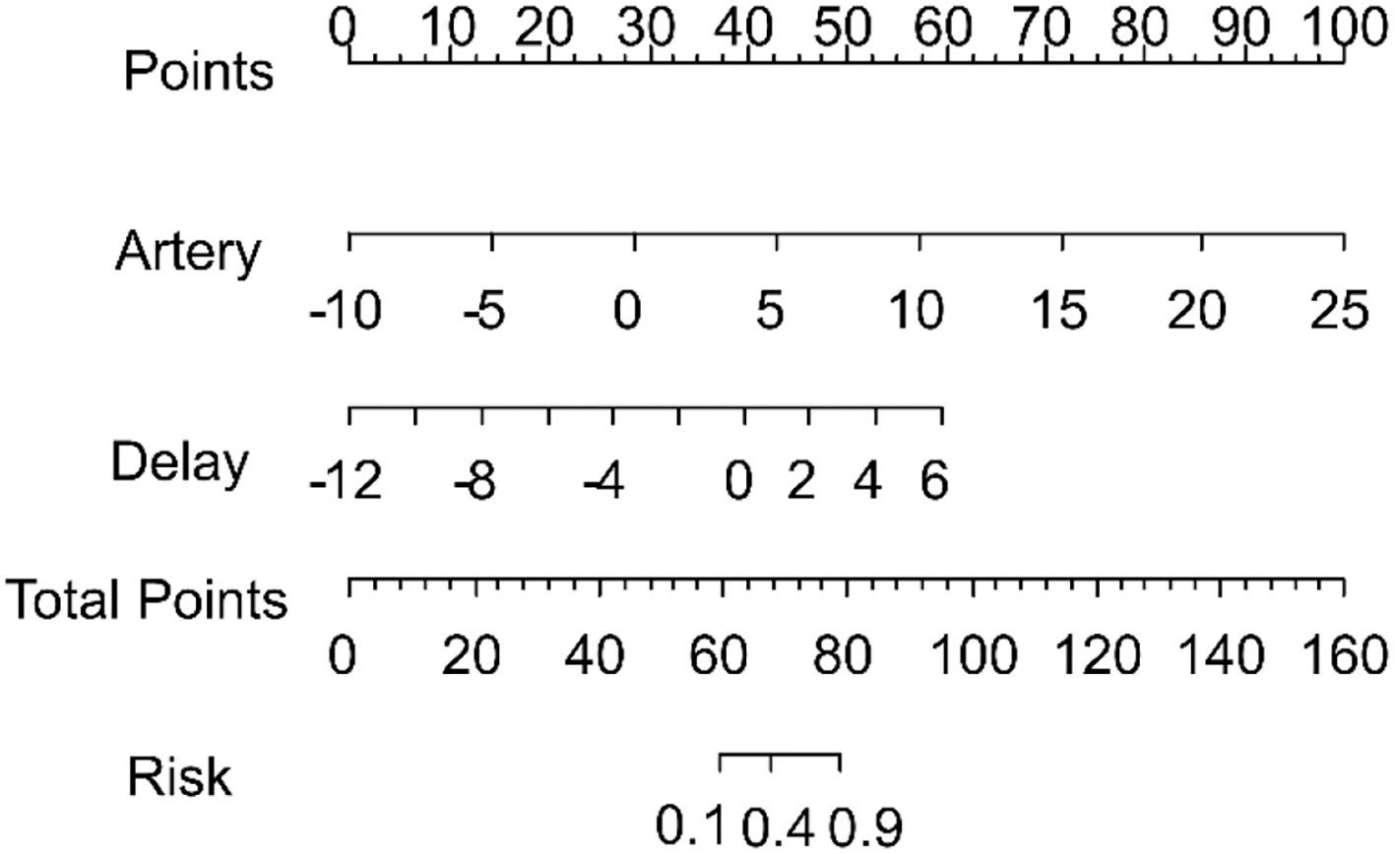
Nomogram combining the radiomics signature from the arterial and delay phases for predicting microvascular invasion. The total score was calculated, and the risk of MVI is shown to go up in the x-axis.

The scores obtained from the clinical model and the four radiomics models were incorporated into a backward stepwise multivariate logistic regression to construct a combined model. After the stepwise backward screening, the radiomics score obtained from the artery and delay models were preserved. The nomograms combining the significant independent predictors of MVI are shown in Figure 6.

The combined model was able to more accurately predict MVI. The ROC curve for our nomogram is shown in Figure 5. The nomogram exhibited the best performance of all models with an AUC of 0.83 (95% CI: 0.74–0.93). DeLong test showed a significant difference between the combined and venous models (p < 0.05). The results of 10 times 10 folds cross validation of the validation set are shown in Supplementary Table 5.

## Discussion

Radiomics is used to extract hundreds of quantitative feature parameters through a computer algorithm to improve the predictive performance of medical images (16). In this retrospective study, we extracted 12 radiomics features associated with MVI of HCC from the non-enhanced phase and from standard triphasic phase images respectively, and then selected these data for radiomics score construction. A total of 48 radiomics features were the optimal textural parameters, which were significantly different between MVI-negative and MVI-positive groups. The wavelet transform displayed the most frequent both in non-enhanced, portal venous and equilibrium phases, meanwhile Log is the most frequent one in arterial phase. The radiomics features showing the best discriminative performance in predicting MVI of each phase are as follow: the AUC of glszm. Zone Percentage based on non-enhanced phase was 0.74, which measures the coarseness of the texture; the AUC of glrlm. Long Run High Gray Level Emphasis based on arterial phase was 0.75, which measures the joint distribution of long run lengths with higher gray-level values; the AUC of ngtdm.Strength based on portal venous phase was 0.70, which is a measure of the primitives in an image; the AUC of shape. Maximum 2D Diameter Column based on equilibrium phase was 0.70, which is defined as the largest pairwise Euclidean distance between tumor surface mesh vertices in the row-slice plane. In these 48 texture parameters, which generated from GLSZM and GLRLM were the most frequently significant difference between the two groups (17–22). The GLSZM and GLRLM both reflect heterogeneity of the image, which can be attributed to the more heterogeneity of MVI-positive group (including more blood vessels with abnormal hyperplasia, more necrosis due to fast tumor growth, and more uneven internal structure of tumor).

We developed radiomics signature models based on non-enhanced, arterial, portal venous, and equilibrium phases capable of predicting MVI, with AUCs of 0.75, 0.79, 0.73, and 0.80, respectively. Our results indicate that the radiomics signatures of standard triphasic CT achieved good performance at all CT phases. These findings are similar to those of Zheng et al. (23), who suggested that a radiomics signature derived from CT images could predict MVI, with an AUC of 0.80 in tumors < 5 cm. In addition, the artery and delay models performed better than the plain and venous models. This result differed from previous studies (24, 25), which suggested that radiomics signatures based on the portal venous phase perform better than those based on the arterial or equilibrium phase. Such inconsistencies might be associated with the presence of selection bias and the inclusion criteria used in our radiomics analysis.

In addition to this radiomics analysis, we evaluated clinical and CT imaging factors. We found that tumor diameter and pathological grade were independent variables associated with MVI. Previous studies (17, 26) also found that tumor size was an independent factor for predicting survival of patients with HCC; this was consistent with our results. We observed the same trend in E-S grades with MVI likelihood. Therefore, we constructed a clinical model including tumor diameter and pathological grades that could discriminate MVI-positive HCC with an AUC of 0.77. Some studies have indicated that serum AFP levels and non-smooth tumor margins are more frequently found in MVI-positive HCC cases (5, 6, 27). Similarly, other studies that developed radiomic signatures using tumor boundary difference and hypointense halo could also predict MVI (5, 28). Interestingly, in our study, multinodular fusion and serum AFP level were also significant in univariate analysis, but they each lost their significant association with MVI in multivariate analysis.

Furthermore, we developed and validated a nomogram as a surrogate biomarker of MVI in HCC, which combined the above models. A nomogram is an effective and accurate model for visualizing regression equations because it can establish scoring standards based on the regression coefficients of all independent variables (13). It transforms the complex regression equation into a simple, visual graph and has been proposed as a new standard method. Recently, it has been widely used as a prognostic tool for many tumor types, such as liver fibrosis (14), hepatocellular carcinoma (27), lung cancer (29) and thyroid cancer (30). Our combined model exhibited the best predictive performance with an AUC of 0.83, and it discriminated MVI better than the clinical and radiomics signature models that used a single CT phase; this indicates that our nomogram can provide additional prognostic and biological information, consistent with previous studies (24, 27, 31). Recent studies have also revealed that when combining clinical, laboratory, semantic, and radiomic signatures, the resultant model has better predictive power than a single one (17, 32). One of these studies (17) showed improved model performance after combining clinical risk factors. Conversely, the other study (32) showed that a clinical-radiomics model had an AUC of 0.835 when comparing the single model (AUC of 0.734 and 0.783) in predicting MVI. In addition, Xu et al. (31) constructed a combined model which combined radiomic signatures with clinical parameters, including AFP, with an accuracy of 82.8% (AUC = 0.889). Furthermore, Zhang et al. (25) demonstrated a multi-disciplinary team-like radiomics fusion model can predict MVI status in HCC. In addition, some studies have used magnetic resonance (MR)-based radiomics to construct a nomogram for predicting MVI (27, 33–35).

Our findings point to the likelihood that a combined model has better performance than any single model. Therefore, we suggest that a combined model should be proposed for use in texture analysis. In this regard, the nomogram that we developed could provide a straightforward, convenient, useful, and robust method for personalized prediction of MVI after aspiration biopsy and could be a useful tool for guiding subsequent personalized treatment such as surgery or TACE.

Previous studies have indicated that differences in image acquisition parameters such as scanning equipment, image resolution, signal-to-noise ratio, reconstruction algorithms, and contrast agent injection rates could influence CT texture quantification (21, 36). We carefully considered the potential impact of the above factors on repeatability, and all participants included in this study were thus scanned using the same equipment, scanning parameters, reconstruction algorithms, and contrast injection rate.

However, this study has some limitations. First, this was a single-center retrospective study, with a small number of participants (*n* = 82). Second, MVI was only grouped into negative or positive and was not considered in the MVI 1/2 group. Further studies with larger samples are required to establish a prediction model for MVI grade and to improve the model's overall performance.

In conclusion, the results of the present study suggest that standard triphasic CT scans combined with a radiomic signature can be used for preoperative prediction of MVI in HCC; all of the tested models had good diagnostic performance, with the established nomogram having the best performance among all six of the models described here. A nonogram such as the one we developed for MVI prediction could prove to be a highly useful tool for guiding subsequent personalized treatment.

## Data Availability Statement

The raw data supporting the conclusions of this article will be made available by the authors, without undue reservation.

## Ethics Statement

The studies involving human participants were reviewed and approved by the Institutional Review Board of the Second Affiliated Hospital of Anhui Medical University, which waived the need for individual informed consent.

## Author Contributions

WY, SY, RZ, and JB: conceptualization, writing—review and editing. WY, SY, YG, WF, and YW: methodology and formal analysis. LX, KG, YW, and YZ: resources. WY and SY: writing and original draft preparation. WY, SY, and JB: revising. All authors have read and agreed to submit the manuscript.

## Funding

This work was equally supported by the Natural Science Foundation of China (32000750 and 51901001), the Anhui Provincial Natural Science Foundation (2008085QH369, 2008085MF221), the Natural Science Foundation of Anhui Medical University (2019xkj031), the Clinical Research Training Program of the Second Affiliated Hospital of Anhui Medical University (2020LCYB05), the Research Fund of the Anhui Institute of Translational Medicine (2021zhyx-C45), the Basic and Clinical Collaborative Research Improvement Project of Anhui Medical University (2020xkjT020), the School Foundation of Anhui Medical University (2019xkj016), Grants for Scientific Research of BSKY (XJ201907) from Anhui Medical University, and Scientific Research Improvement Project of Anhui Medical University (2021xkjT018).

## Conflict of Interest

YG was employed by GE Healthcare China. The remaining authors declare that the research was conducted in the absence of any commercial or financial relationships that could be construed as a potential conflict of interest.

## Publisher's Note

All claims expressed in this article are solely those of the authors and do not necessarily represent those of their affiliated organizations, or those of the publisher, the editors and the reviewers. Any product that may be evaluated in this article, or claim that may be made by its manufacturer, is not guaranteed or endorsed by the publisher.
