# Supplementary material for: Computed Tomography Radiomics-Based Prediction of Microvascular Invasion in Hepatocellular Carcinoma
Source: Front Med (Lausanne). 2022 Mar 24;9:819670. doi: 10.3389/fmed.2022.819670 (PMC8987588; doi:10.3389/fmed.2022.819670)
Supplement: Supplementary file 1 [file Table_1.docx]

Supplementary Table 1 the details the cohort demographics and tumor characteristics

| Variables | Sample(n=82) | MVI-negative (n=33) | MVI-positive (n=49) | P-value |
| --- | --- | --- | --- | --- |
| Clinical risk factors |  |  |  |  |
| Gender |  |  |  | 0.520 |
| male | 65 | 25(75.76%) | 40(81.63%) |  |
| female | 17 | 8(24.24%) | 9(18.37%) |  |
| Age(year) | 82 | 56.00(53.40, 65.00) | 57.00(53.00, 64.30) | 0.813 |
| serum AFP level | 82 | 15.80(3.74, 256.42) | 409.20(4.36, 1392.10) | 0.013 |
| Hepatitis B infection |  |  |  | 0.834 |
| Absent | 12 | 5(15.15%) | 7(14.29%) |  |
| Present | 70 | 28(84.85%) | 42(85.71%) |  |
| Liver cirrhosis |  |  |  | 0.665 |
| Absent | 30 | 13(39.39%) | 17(34.69%) |  |
| Present | 52 | 20(60.61%) | 32(65.31%) |  |
| E-S grade |  |  |  | 0.004 |
| I | 6 | 4(12.12 %) | 2(4.08 %) |  |
| II | 36 | 23(69.70 %) | 13(26.53 %) |  |
| III | 36 | 5(15.16 %) | 31(63.27 %) |  |
| IV | 4 | 1(3.03 %) | 3(6.12%) |  |
| CT imaging features |  |  |  |  |
| Diameter(cm) | 82 | 4.10(2.17, 6.30) | 6.50(4.07, 10.26) | 0.002 |
| Tumor margin |  |  |  | 0.441 |
| Smooth | 38 | 17(51.52%) | 21(42.86%) |  |
| Non-smooth | 44 | 16(48.48%) | 28(57.14%) |  |
| Capsule |  |  |  | 0.653 |
| Absent | 50 | 20(60.61%) | 30(61.22%) |  |
| Incomplete | 25 | 9(27.27%) | 16(32.65%) |  |
| Complete | 7 | 4(12.12%) | 3(6.12%) |  |
| Locally convex nodules |  |  |  | 0.499 |
| Absent | 41 | 18(54.55%) | 23(46.94%) |  |
| Present | 41 | 15(45.45%) | 26(53.06%) |  |
| Multinodular fusion |  |  |  | 0.033 |
| Absent | 59 | 28(84.85%) | 31(63.27%) |  |
| Present | 23 | 5(15.15%) | 18(36.73%) |  |
| TTPVI |  |  |  | 0.441 |
| Absent | 38 | 17(51.52%) | 21(42.86%) |  |
| Present | 44 | 16(48.48%) | 28(57.14%) |  |
| PVTT |  |  |  | 0.980 |
| Absent | 62 | 25(75.76%) | 37(75.51%) |  |
| Present | 20 | 8(24.24%) | 12(24.49%) |  |

Supplementary Table 2 the number of features retained by each feature selection method

| Phase | ICC>0.75 | Univariate analysis  (p<0.05) | Univariate logistic regression  (p<0.05) |
| --- | --- | --- | --- |
| Non-enhanced | 727 | 113 | 105 |
| Arterial | 884 | 261 | 234 |
| Portal venous | 612 | 102 | 92 |
| Equilibrium | 933 | 97 | 84 |

Supplementary Table 3 performance of selected features with high correlation

| Phase | Image type | Variable name | threshold | Accuracy | Sensitivity | Specificity |
| --- | --- | --- | --- | --- | --- | --- |
| Non-enhanced | wavelet.HHL | glszm.ZonePercentage | -0.17 | 0.73 | 0.80 | 0.64 |
|  | wavelet.HHL | glrlm.ShortRunEmphasis | 0.24 | 0.70 | 0.73 | 0.64 |
|  | wavelet.HHL | gldm.DependenceNonUniformityNormalized | 0.66 | 0.57 | 0.33 | 0.94 |
|  | wavelet.HHH | ngtdm.Busyness | -0.66 | 0.72 | 0.86 | 0.52 |
|  | wavelet.LLH | glszm.ZoneEntropy | -0.15 | 0.72 | 0.78 | 0.64 |
|  | wavelet.LLH | glszm.GrayLevelNonUniformityNormalized | -0.20 | 0.62 | 0.53 | 0.76 |
|  | lbp.3D.k | glszm.LowGrayLevelZoneEmphasis | -0.16 | 0.68 | 0.86 | 0.42 |
|  | lbp.3D.k | glrlm.GrayLevelVariance | 0.16 | 0.68 | 0.82 | 0.48 |
|  | lbp.3D.m1 | glszm.SmallAreaLowGrayLevelEmphasis | -0.19 | 0.74 | 0.88 | 0.55 |
|  | log.sigma.2.0.mm.3D | glrlm.RunLengthNonUniformity | -0.49 | 0.70 | 0.71 | 0.67 |
|  | log.sigma.3.0.mm.3D | glszm.GrayLevelNonUniformity | -0.43 | 0.72 | 0.78 | 0.64 |
|  | original | ngtdm.Coarseness | -0.18 | 0.73 | 0.82 | 0.61 |
| Arterial | log.sigma.2.0.mm.3D | gldm.LargeDependenceHighGrayLevelEmphasis | -0.12 | 0.65 | 0.49 | 0.88 |
|  | log.sigma.2.0.mm.3D. | firstorder.90Percentile | -0.13 | 0.65 | 0.63 | 0.67 |
|  | log.sigma.2.0.mm.3D. | firstorder.Skewness | -0.04 | 0.62 | 0.51 | 0.79 |
|  | log.sigma.3.0.mm.3D | glrlm.LongRunHighGrayLevelEmphasis | -0.40 | 0.74 | 0.84 | 0.61 |
|  | log.sigma.3.0.mm.3D | gldm.LargeDependenceLowGrayLevelEmphasis | -0.48 | 0.67 | 0.59 | 0.79 |
|  | log.sigma.3.0.mm.3D | glcm.Correlation | -0.59 | 0.74 | 1.00 | 0.36 |
|  | wavelet.HHH | glszm.ZoneEntropy | -0.70 | 0.74 | 0.90 | 0.52 |
|  | wavelet.HHL | glszm.ZonePercentage | -0.34 | 0.70 | 0.65 | 0.76 |
|  | wavelet.LLL | glrlm.RunLengthNonUniformity | -0.68 | 0.72 | 0.86 | 0.52 |
|  | lbp.3D.m1 | firstorder.Skewness | 1.04 | 0.72 | 1.00 | 0.30 |
|  | lbp.3D.m2 | firstorder.90Percentile | 0.33 | 0.61 | 0.53 | 0.73 |
|  | original | glcm.MCC | 0.28 | 0.65 | 0.55 | 0.79 |
| Portal Venous | wavelet.HHH | ngtdm.Strength | -0.05 | 0.72 | 0.86 | 0.52 |
|  | wavelet.HHL | gldm.DependenceEntropy | 0.86 | 0.66 | 0.88 | 0.33 |
|  | wavelet.HLL | glszm.ZonePercentage | -0.26 | 0.67 | 0.69 | 0.64 |
|  | wavelet.HLL | gldm.DependenceNonUniformityNormalized | -0.27 | 0.66 | 0.73 | 0.55 |
|  | wavelet.HLH | gldm.DependenceVariance | 0.04 | 0.70 | 0.78 | 0.58 |
|  | wavelet.LHL | glszm.GrayLevelNonUniformityNormalized | -0.19 | 0.61 | 0.51 | 0.76 |
|  | wavelet.LLH | glszm.ZoneEntropy | -1.20 | 0.68 | 0.94 | 0.30 |
|  | log.sigma.2.0.mm.3D. | firstorder.Median | 0.14 | 0.70 | 0.78 | 0.58 |
|  | log.sigma.2.0.mm.3D | glcm.Idn | -0.01 | 0.65 | 0.69 | 0.58 |
|  | log.sigma.3.0.mm.3D | glrlm.RunVariance | -0.26 | 0.68 | 0.71 | 0.64 |
|  | lbp.3D.k | glrlm.RunVariance | -0.24 | 0.65 | 0.63 | 0.67 |
|  | original | shape.Maximum2DDiameterColumn | -0.46 | 0.72 | 0.78 | 0.64 |
| Equilibrium | wavelet.HHL | glszm.ZonePercentage | -0.12 | 0.71 | 0.82 | 0.55 |
|  | wavelet.HHH | glrlm.RunVariance | 0.50 | 0.67 | 0.57 | 0.82 |
|  | wavelet.LLH | gldm.LowGrayLevelEmphasis | 0.19 | 0.67 | 0.80 | 0.48 |
|  | wavelet.LLH | glszm.SmallAreaLowGrayLevelEmphasis | 0.16 | 0.68 | 0.76 | 0.58 |
|  | wavelet.LHL | glszm.SmallAreaEmphasis | -0.83 | 0.68 | 0.90 | 0.36 |
|  | wavelet.LLL | glrlm.LongRunLowGrayLevelEmphasis | 1.09 | 0.67 | 0.96 | 0.24 |
|  | original | ngtdm.Coarseness | -0.21 | 0.71 | 0.76 | 0.64 |
|  | original | shape.Maximum2DDiameterColumn | -0.83 | 0.73 | 0.92 | 0.45 |
|  | original | glrlm.RunEntropy | -0.35 | 0.66 | 0.71 | 0.58 |
|  | lbp.3D.k | glszm.GrayLevelVariance | 0.13 | 0.71 | 0.90 | 0.42 |
|  | lbp.3D.k | glcm.DifferenceEntropy | -0.02 | 0.62 | 0.61 | 0.64 |
|  | lbp.3D.k | glszm.SmallAreaHighGrayLevelEmphasis | -0.39 | 0.68 | 0.92 | 0.33 |

Supplementary Table 4 The AUC of optimal texture parameters based on each phase of CT images

| Phase | Image type | Variable | AUC | 95% confidence interval |
| --- | --- | --- | --- | --- |
| Non-enhanced | wavelet.HHL | glszm. ZonePercentage | 0.74 | 0.63-0.86 |
| Arterial | log.sigma.3.0.mm.3D | glrlm. Long Run High Gray Level Emphasis | 0.75 | 0.64-0.84 |
| Portal venous | wavelet.HHH | ngtdm.Strength | 0.70 | 0.58-0.83 |
| Equilibrium | original | shape.Maximum 2D Diameter Column | 0.70 | 0.58-0.82 |

Supplementary Table 5 the Specificity, Sensitivity, Accuracy, PPV, NPV of each model

| Model name | Specificity | Sensitivity | Accuracy | Pos.pred.value | Neg.pred.value |
| --- | --- | --- | --- | --- | --- |
| Clinical | 0.75 | 0.78 | 0.77 | 0.86 | 0.64 |
| Plain | 0.58 | 0.84 | 0.73 | 0.75 | 0.70 |
| Artery | 0.76 | 0.71 | 0.73 | 0.81 | 0.64 |
| Venous | 0.55 | 0.92 | 0.77 | 0.75 | 0.82 |
| Delay | 0.67 | 0.90 | 0.80 | 0.80 | 0.81 |
| Combined | 0.91 | 0.80 | 0.83 | 0.96 | 0.64 |
